# Perceptual anchoring effects: Evidence of response bias and a change in estimates sensitivity

**DOI:** 10.1002/brb3.3254

**Published:** 2023-10-13

**Authors:** Teresa Garcia‐Marques, Alexandre Fernandes

**Affiliations:** ^1^ William James Center for Research ISPA—Instituto Universitário, Lisbon, Portugal

**Keywords:** absolute/relative estimates, anchoring effects, psychophysics, signal detection approach

## Abstract

**Introduction:**

People's estimates of perceptual quantities are commonly biased by the contextual presence of other quantities (like numbers). In this study, we address assimilation anchoring effects (approximation of real quantities to contextual quantities) that occur for visually displayed proportions, defining a new methodological setting for the effect.

**Method:**

Similar to classic approaches, we asked participants across several trials whether the display contained a feature in a proportion higher or lower than “a randomly selected value” (relative judgments), and then estimated the feature proportions (absolute judgments). Across all trials, we presented seven anchors ranging from .20 to .80, each with a visually displayed representation of the same seven proportions (49 judgments in total). This allowed for a psychophysical approach to individual estimates and signal detection indexes, providing new insights into how the anchoring effect is generated in this setting.

**Results:**

Our findings suggest that anchoring effects occur both as a bias (changes in response criteria) and as a change in the ability to discriminate stimuli (affecting sensitivity indexes). Moreover, anchors modulate the level of stimuli features for which estimates were more uncertain. Finally, our results indicate that anchor effects occur immediately in the first phase of the two‐phase paradigm, leading to the availability of values for supporting absolute estimates.

**Conclusion:**

By using a psychophysical approach to the anchoring effects, for the first time, we could clarify that this effect is the result of both bias and changes in the ability to discriminate quantity.

## Graphical abstract

We provide evidence of the perceptive anchoring effect. It occurs as a result of response bias and a change in estimate sensitivity. Anchors modulate the level of stimulus features for which estimates are more uncertain and more prone to anchoring effects.

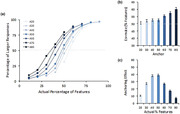

## 1 INTRODUCTION

Assimilative anchoring effects, as defined by Tversky and Kahneman (1974), refer to the tendency of magnitude estimates to align with previously presented values. This effect is regarded as a bias in our estimation process induced by the initial exposure to a specific quantity or numerical value. For example, if we are initially exposed to a value of 1000 and then asked to estimate the value of a certain object, our estimates are likely to be higher compared to if we were exposed to a lower numerical value beforehand. These effects were initially observed in relation to estimates of retrieved memory events.

In this study, we examine assimilation anchoring effects that arise when visually displayed proportions are perceived, utilizing a psychophysical approach and signal detection indexes to investigate their underlying mechanisms. The presence of anchoring effects on directly perceived quantities was previously demonstrated by LeBoeuf and Shafir (2006), who found that estimates of physical stimuli's magnitude are influenced by the presence of physical anchors. Subsequent research by Alards‐Tomalin et al. (2015) and Lange borg and Eriksson (2016) further clarified that anchors have the potential to distort estimates when perceptual stimuli are present, aligning with the hypothesis of assimilative bias. However, the interpretation of the perceptual anchoring effect is challenged due to its occurrence in a setting where numeric perceptual information is directly available. This challenges its characterization solely as a response generation bias. Instead, in this context, anchors can interfere with the estimation process through an alternative route. They can potentially affect the perceptual system, altering the way stimuli are experienced and influencing individuals' sensitivity to differences between stimuli. Therefore, we aim to explore these two possibilities by examining the two components of a signal detection approach, namely response bias and sensitivity, in individual estimations of visual quantities.

In the following section, we provide a concise overview of the literature concerning memory and perceptual anchoring effects, as well as how these effects have been investigated. Subsequently, we introduce a novel experimental paradigm that extends the conventional two‐stage procedure to multiple trials. This new paradigm allows for systematic variations of both anchor and target quantities, enabling a comprehensive psychophysical approach to the study of perceptual anchoring effects guiding our hypothesis.

## 2 MEMORY AND PERCEPTUAL ANCHORING EFFECTS

Evidence of an anchoring effect was first provided by Tversky and Kahneman (1974) using a two‐stage experimental paradigm. In the first stage, researchers ostensibly drew a number from a roulette wheel and asked participants whether the percentage of African countries in the United Nations was below or above this number. In the second stage, they require participants to provide a concrete absolute estimate for this percentage. The results showed that the initial irrelevant provided value (the anchor) affected the percentage provided by the participants. Those participants who received a low value made lower absolute estimates than those who received a high value. Subsequent research has repeatedly shown that simply considering a value (with no relevance) before making judgments biases individuals’ judgments by assimilating the value to the anchor (see Chapman & Johnson, 2002; Epley & Gilovich, 2006; Wilson et al., 1996).

Since the pioneering work of Tversky and Kahneman (1974), anchoring has been studied in cognitive and social psychology as an assimilative bias exerted over our quantitative judgments that rely on information retrieved from or generated in memory. That is, anchoring effects were mostly identified for “known” or “imaginary” events (see Furnham & Boo, 2011). These effects were found to occur for factual questions, such as the number of countries represented in the United Nations or the size of the Mississippi River (e.g., Epley & Gilovich, 2001), and for more subjective expectations, such as legal judgments (e.g., Englich & Mussweiler, 2001), numbers of migrants to be accepted (Lalot et al., 2019), the likelihood of purchase behaviors (e.g., Ariely et al., 2003), and self‐efficacy (Cervone & Peake, 1986) and self‐confidence judgments (Carroll et al., 2009).

However, long preceding the interest of judgment and decision‐making researchers, anchoring effects have been explored in psychophysics as a contextual effect (e.g., Cohen, 1937; Volkmann, 1951). In those studies, the number that biases individuals’ magnitude estimates was offered within the task instructions, in a previous exposure task, or as a magnitude of another stimulus presented in the context. Changes in these quantities promoted changes in the magnitude of the perceptive estimation, but usually in the opposite direction (e.g., Aagten‐Murphy & Burr, 2016; Behar & Bevan, 1961; Brown, 1953; Campbell et al., 1958); higher anchors led to underestimation while lower anchors led to overestimation, and approximately 50% had no effect. This could define perceptual anchoring as a contrast effect in opposition to those relying on memory retrieval processes. However, we have also observed a similar effect on anchoring in memory events, suggesting that a contrast effect may exceptionally emerge when extreme anchors are used (see, e.g., Mussweiler, 2002; Schwarz & Bless, 1992; Sherif et al., 1958; also see Strack & Mussweiler, 1997), and several studies also report evidence of an assimilation effect within a perceptive setting, at least when they used less extreme anchors (e.g., Parducci & Marshall, 1962; Sherif, et al., 1958).

More recently, LeBoeuf and Shafir (2006) revisited this topic by approaching anchoring effects at a perceptive level. Their results show evidence of assimilative anchoring effects using an experimental task inspired by the Tversky and Kahneman procedures, with physical stimuli resembling those of psychophysical studies (and defined across disparate modalities and dimensions). In their studies, participants estimated the length, weight, or loudness of a target, either by extending a small physical anchor or by diminishing a large anchor. The results show evidence of an assimilation pattern, also matching the idea that the required adjustment of the quantity defined by the anchor was always insufficient. Additional research corroborates that estimates of physical quantities are sensitive to anchoring within an assimilation pattern. Alards‐Tomalin et al. (2015) show that anchoring affects judgments of loudness (but only when the anchor number was actively held in short‐term memory), and Langeborg and Eriksson (2016) show numeric anchoring in a target age estimation and in the estimation of the number of objects that were perceived within a glass.

The assumption that the identification of anchoring occurring at a perceptual level was an extension of the impact of the anchor that had been identified in other paradigms led these authors to simply assume that it was a bias and to rely on its explanation in the same mechanisms that were assumed to promote the memory anchoring effect. For instance, LeBoeuf and Shafir (2006) explain that the perceptive anchor effects using the initial theoretical view are related to an insufficient adjustment of an initial judgment that uses the anchor as a baseline. This explanation assumes that the effect was a bias promoted by the use of an anchoring‐and‐adjustment heuristic (Tversky & Kahneman, 1974; see Chapman & Johnson, 2002; Epley & Gilovich, 2004). An alternative available explanation would be to assume that an anchor does not bias individuals’ responses but instead promotes a distortion of the scale where the quantity is to be mapped (Frederick & Mochon, 2012). Other alternative explanations of anchoring effects that stated them as a bias better match the effects of an anchor over memory than over perceptive responses. These explanations rely on research with factual and imaginary stimuli that show evidence of confirmatory hypothesis testing (Chapman & Johnson, 1994; Strack & Mussweiler, 1997) and of a numeric priming process (Oppenheimer et al., 2008; Wong & Kwong, 2000) that occurred concomitantly with anchoring bias. These effects support the idea that anchors bias access to relevant information in memory, and turning memory accessibility is not only a relevant mechanism to support such estimation processes but also the main source of bias.

Thus, one can assume that the perceptual anchoring effect simply extends memory anchoring effects and that the same set of explanations accounts for both effects. However, and despite being parsimonious, this assumption must be held with caution and subject to criticism because, as the experts in the field repeatedly claimed, anchoring is likely not a single phenomenon with a single explanation but rather an outcome that can occur through multiple processes (e.g., Epley & Gilovich, 2006; LeBoeuf & Shafir, 2006, 2009; Russo, 2010; Simmons et al., 2010; Wegener et al., 2010; Wong & Kwong, 2000). Anchors may interfere with different cognitive processes or with their subprocesses leading to no unified explanations to match all different types of anchoring effects.

As we stated in the introduction of this paper, contrary to the memory anchoring effect, the perceptual anchoring effect occurs in a setting where the information about which a numerical judgment (the stimulus) is made is also perceptively available. This suggests that the anchors can interfere with the estimation process through different routes. One route is by interfering with the perceptive system, promoting changes in how stimuli are experienced affecting the individuals’ degree of sensitivity to differences between stimuli. Another route is by interfering with the response generation, directly biasing the response provided. These two routes may co‐occur and they match the two decision components identified by signal detection theory, sensitivity, and response bias.

### 2.1 Perceptual anchoring effects within a psychophysical approach

Humans’ visual system is sensitive to similarities and differences between percepts, sustaining their cognitive categorization, and providing access to their summary statistical information across different perceptive dimensions (see Whitney & Leib, 2018 for a revision). The human capacity to rapidly estimate quantities available through perception (see Anobile et al., 2016; Castaldi et al., 2020; Cicchini et al., 2016) allows humans to use these estimates as a natural dimension for discriminating events from each other.

A psychophysical approach to those estimations anchors on the definition of the relationship between physical stimuli and the estimation they produce. It allows us to define at an individual level the function that associates different levels of the percepts with different estimations to find their regularities. The specific function defined is dependent upon the dimension of the stimuli that are modulated. Aiming to approach perceptive assimilation anchor effects within a psychophysical approach, we need to define the dimension of that stimulus to be modulated. This quantity can go from numerosity to more continuous quantities, either presented as absolute or relative quantities (i.e., proportions). Thus, when focusing on proportions, we may consider, for instance, the number of one type of marble in a jar (numerosity) or the volume of water in a glass (continuous quantity).

To develop an experimental task that allows addressing perceptual anchoring effects occurring in psychophysical function, we will rely on proportions. Proportion estimates, being relative quantities, offer a direct setting for integrating estimates of different settings and dimensions and thus facilitate the development of an experimental task that aims to address anchoring effects at a perceptive level. Most of the features that characterize proportion estimates are similar to those that affect other magnitude estimates even though proportion estimates rely on more complex reasoning and displays (with one type of element being considered relative to the other or the whole; see Hollands & Dyre, 2000). However, there are also typical distortions in the psychophysical function of proportion estimates. Although some of the first psychophysical approaches to proportion estimates established a linear relationship with actual proportions (Philip, 1947; Shuford, 1961), other and more recent research identified biases promoted by the actual proportion extreme values, leading a nonlinear model to fit this relationship better (e.g., Stevens & Galanter, 1957; Zhang & Maloney, 2012). Small numbers or proportions (< .20) tend to be overestimated, whereas large numbers or proportions (> .80) tend to be underestimated (e.g., Sheridan & Ferrell, 1974; Varey et al., 1990; Wickens, 1992; but see Hollands & Dyre, 2000). Although this pattern of relationship occurs independently of the target corresponding to visual concrete stimuli, memory events, or knowledge‐based estimates (e.g., Landy et al., 2017; Lichtenstein et al., 1978; Tversky & Kahneman, 1992), by focusing it within a psychophysical approach, we can address how anchors promote changes in parameters of this function.

To assess how anchors interfere with perceptual processes, our experimental task is different from memory anchoring paradigms in different ways. First, the quantities to be estimated are perceptive. Second, we systematically vary the properties of a stimulus to be accessed since the participants need to perform several trials where different levels of the property of a stimulus are systematically associated with the same anchor to define the psychophysical function for each participant. Third, we systematically vary the size of the anchor to address how they change each participant's psychophysical function.

The development of an experimental paradigm with these features implies a cover story for an anchor being presented and randomly associated with a stimulus. For this, we use the two‐phase paradigm originally proposed by Tversky and Kahneman (1974) and conduct each trial using this two‐stage structure. In the first stage, participants are asked to make a relative judgment after an anchor is presented. In the second stage, a request for an absolute judgment is made.

The anchoring effects observed in relation to absolute estimates are often considered distortions of the psychophysical function influenced by the anchor. Various approaches to this effect interpret it as a bias and describe it as a shift in the central tendency of the function. However, the change in a central tendency may occur either because the context biases the response tendency and/or because the contextual feature impacts individuals' ability to discriminate perceived quantities (e.g., Morgan & Glennerster, 1991). Previous studies were not able to properly characterize the effect as the assumed bias because the one‐trial experimental paradigm typically used did not allow the studies to characterize the distortion in their single detection components and distinguish the bias from changes in response sensitivity. Contrary to those studies, the experimental paradigm we propose allows the use of *signal detection analysis* (SDT) to separate the two components of external influences. Therefore, the paradigm will allow us to verify if the perceptual anchoring effect is a *pure bias effect*, as the literature has claimed (when referring to memory anchoring effects), or if the anchor impacts response sensitivity to changes in the environment.

As a mathematical theory, SDT modulates how a system detects signals embedded in noise and allows the estimation of two parameters. As such, SDT models can be fit to individuals' psychometric functions and help to determine the parameters that represent a response bias or a change in the functional sensitivity (see below).

There is an additional advantage of the proposed paradigm. Studies that rely on this two‐stage paradigm have only focused on the absolute judgment generated in the second phase and had little consideration for the first judgment, which was only for providing the anchor (see Bahník et al., 2017; Chapman & Johnson, 2002; Epley, 2004). However, Strack and Mussweiler (1997) show that while participants are providing an answer to an initial decision, they may already anticipate the generation of the absolute judgment. The paradigm proposed allows us to better understand the psychological mechanism that leads to anchoring effects by understanding if the mechanism is better detected at the moment the anchor is presented or when the absolute estimate is provided.

### 2.2 The perceptual estimation anchor task

The perceptual estimation anchor task adapts a psychophysical approach to proportion estimates (targets are perceptual displays that vary systematically in their real proportions) in which each trial is defined by two stages that allow anchors to vary systematically. Below, we detail the task features and subsequently the relevant response parameters for analysis.

#### 2.2.1 Task features

**FIGURE 1:**
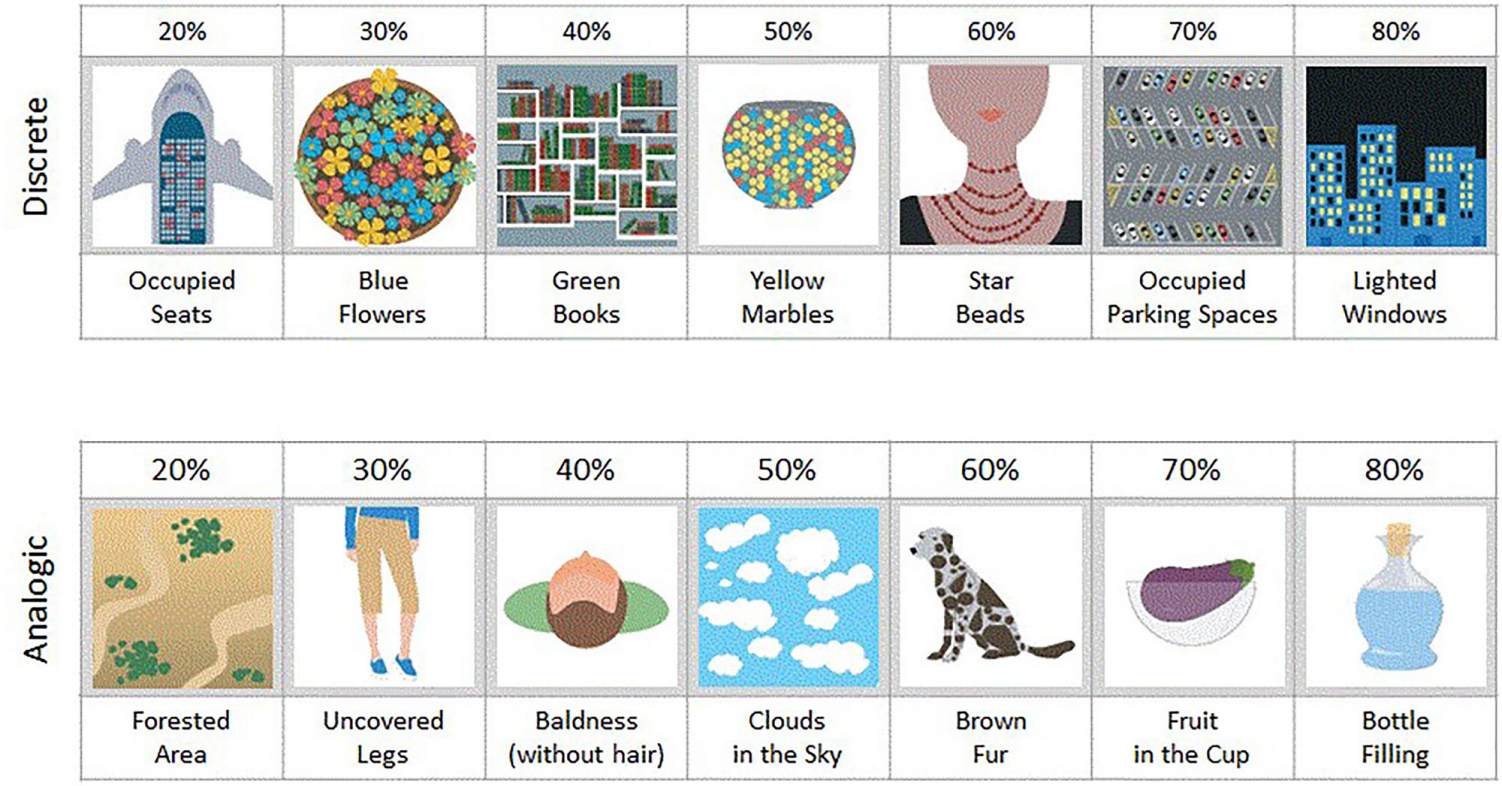
Seven discrete sets (upper panel) and seven continuous sets (bottom panel) of images were used; each set had three or four variants, each presented to participants in all seven different levels of feature percentage. Here are exemplars of each set of images with a particular feature percentage (also as an example).

Participants are asked to perform a series of trials of relative and absolute estimates. Target images (from Varatojo et al., 2022) varying in the proportion with which a specific feature occurs (see Figure 1) were presented randomly across trials. In each trial, participants were presented with a target image and required to first make a classification decision (relative judgment) by comparing the target to a reference point (an anchor) and choose between a “larger than” or “smaller than” decision and, subsequently, to make a numerical estimate of quantity (an absolute judgment; the estimated value that represents the percentage of a target feature). The two estimated reaction times are also of relevance, as detailed below.

Targets and anchors are defined within seven proportions equally spaced with increments of 10% between 20% and 80%. A total of 49 images (X 7 proportions) varying in one feature either discretely (25 images) or continuously (24 images; see Figure 1) support such manipulations. For each anchor value, they allow presenting seven replicas for each of the seven proportions (three to four variants of discrete and continuous stimuli) always using a different image. Random selection of images turns the effects independent of materials.

#### 2.2.2 Analyzing responses and expected anchoring effects using the psychophysical signal detection approach

The *relative estimates* offered in the first phase of the paradigm by each participant are mapped concerning the different levels of the proportion presented for each offered anchor. The mapping uses the constant‐stimuli method (with one standard), which results in a typical sigmoidal function (Macmillan & Creelman, 2004; Schiffman, 2003) that fits a logit function well. It is worth noting that only the distributions involving images with 50% of features to be estimated are unrestricted in terms of their position within the estimates distribution. This lack of constraint allows for the possibility of a symmetric distribution of biases. All other levels of proportions are confined to either the lower or upper level of the distribution. Nevertheless, because the results are complimentary for all other materials, they allow inferring the entire distribution and testing for the effects promoted by the anchors and having the matching between the real proportion and the anchor as the same referent point. Since a different logit function is defined for each anchor, anchor effects are assessed as distortions of those functions. SDT suggests that these distortions can be defined by two different parameters of the function associated with bias and sensitivity: the point of subjective equality (PSE) and the difference limen (DL) (see Macmillan & Creelman, 2004; Schiffman, 2003).

**FIGURE 2:**
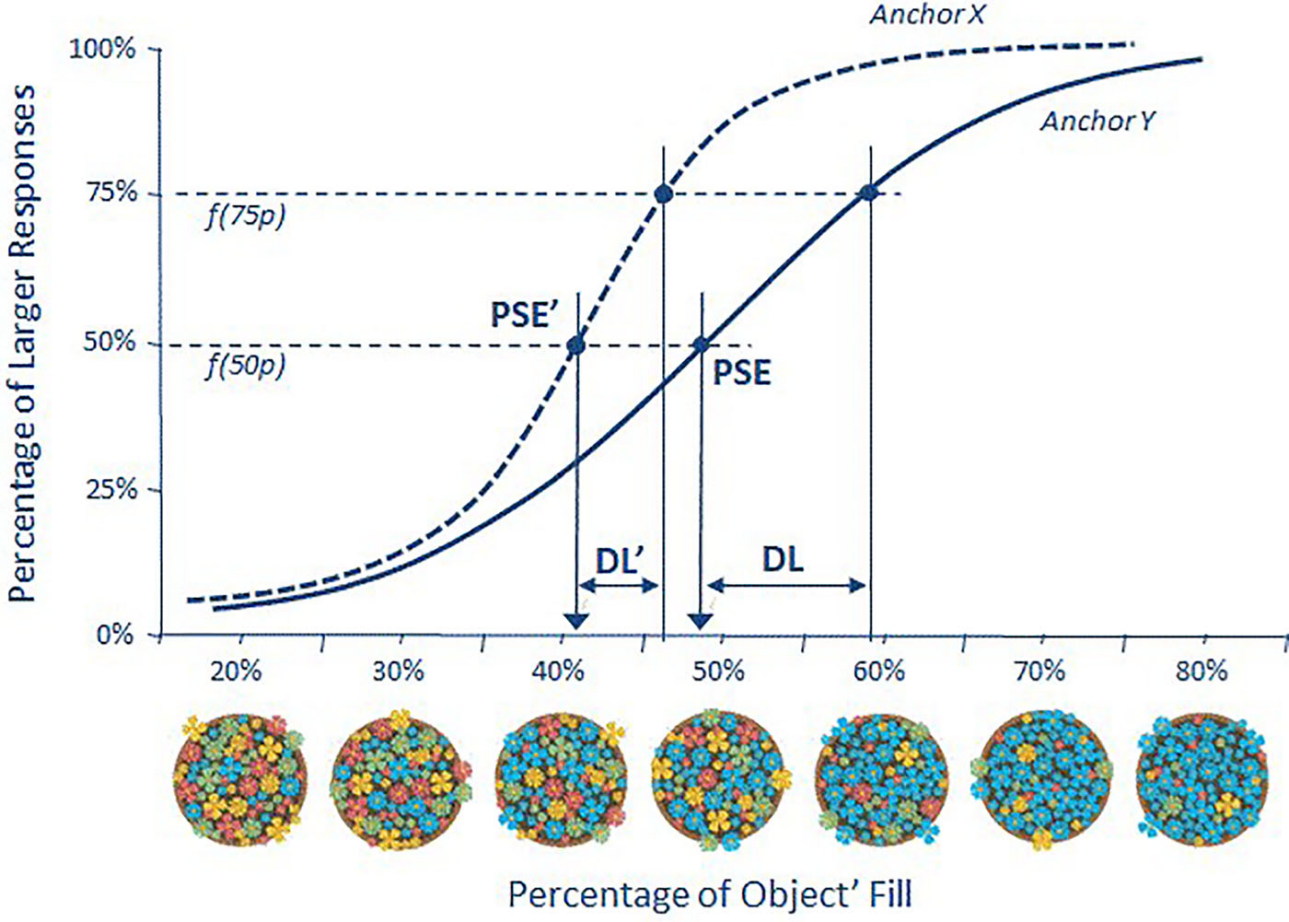
Examples of two logistic functions are used to map individual relative judgments for each level of the actual feature percentage (in the context of hypothetical X and Y anchors). The difference in the (PSE) represents the bias component of the anchoring effect, with lower PSE values indicating a positive bias or a shift in the psychophysical function curve toward higher estimates. The difference in the difference limen (DL) represents the estimated sensitivity component of the anchoring effect, with larger DL values indicating poor sensitivity to the actual proportion of the feature.

Figure 2 shows how anchors can influence both of these components illustrating our hypothesis. It provides the expected logistic functions in the context of higher X and lower Y anchors. The PSE represents the bias or shifting of the psychophysical function curve (with lower PSEs being associated with more positive bias) and the differences for each anchor reflecting the component bias of the anchoring effect. The DL represents the estimated sensitivity to real values of the feature (larger DLs indicating poorer feature discrimination), and differences in the DLs for each anchor represent our hypothesis regarding anchoring effects also having a sensitive component.

The PSE is calculated as the point (of the feature percentage) where the individual provides an equal number of larger and smaller responses (50%), defining the threshold that each individual seems to establish to respond if the estimate is larger or is smaller than the anchor. Larger PSEs indicate higher criteria to respond larger than the standard, and larger PSEs indicate lower estimates (of the feature percentage).

The DL is defined as half the distance between the points (of the feature percentage) associated with 25% and 75% of the larger responses. The DL establishes the difference between quantities that are noticeable by participants such that larger DLs indicate poorer feature sensitivity or discrimination. PSE establishes a negative relationship with the integral of psychophysical function (i.e., the area under the curve), and the DL establishes a negative correlation with the slope of the central part of the curve (Macmillan & Creelman, 2004; Schiffman, 2003).

We may also test our hypothesis based on the *absolute estimates* offered in the second stage for each target magnitude by each participant by using them to define his or her psychophysical function. For each individual, a different function is estimated for each anchor value. Theoretically, given the absence of extreme values, these functions are fit by a linear relationship between the stimulus feature percentage and individuals’ absolute estimates. Different functions are calculated for each anchor always having the matching between the real proportion and the anchor as a reference. Again it is worthwhile to notice that only the distributions of the 50% set of trials are symmetrically restrained in the bottom or upper level of the distribution.

Because our absolute judgments are made on a scale from 0% to 100% and establish a linear relationship with the real magnitude, we can also calculate the SDT indices similarly to the way we calculate them for the relative judgments.

**FIGURE 3:**
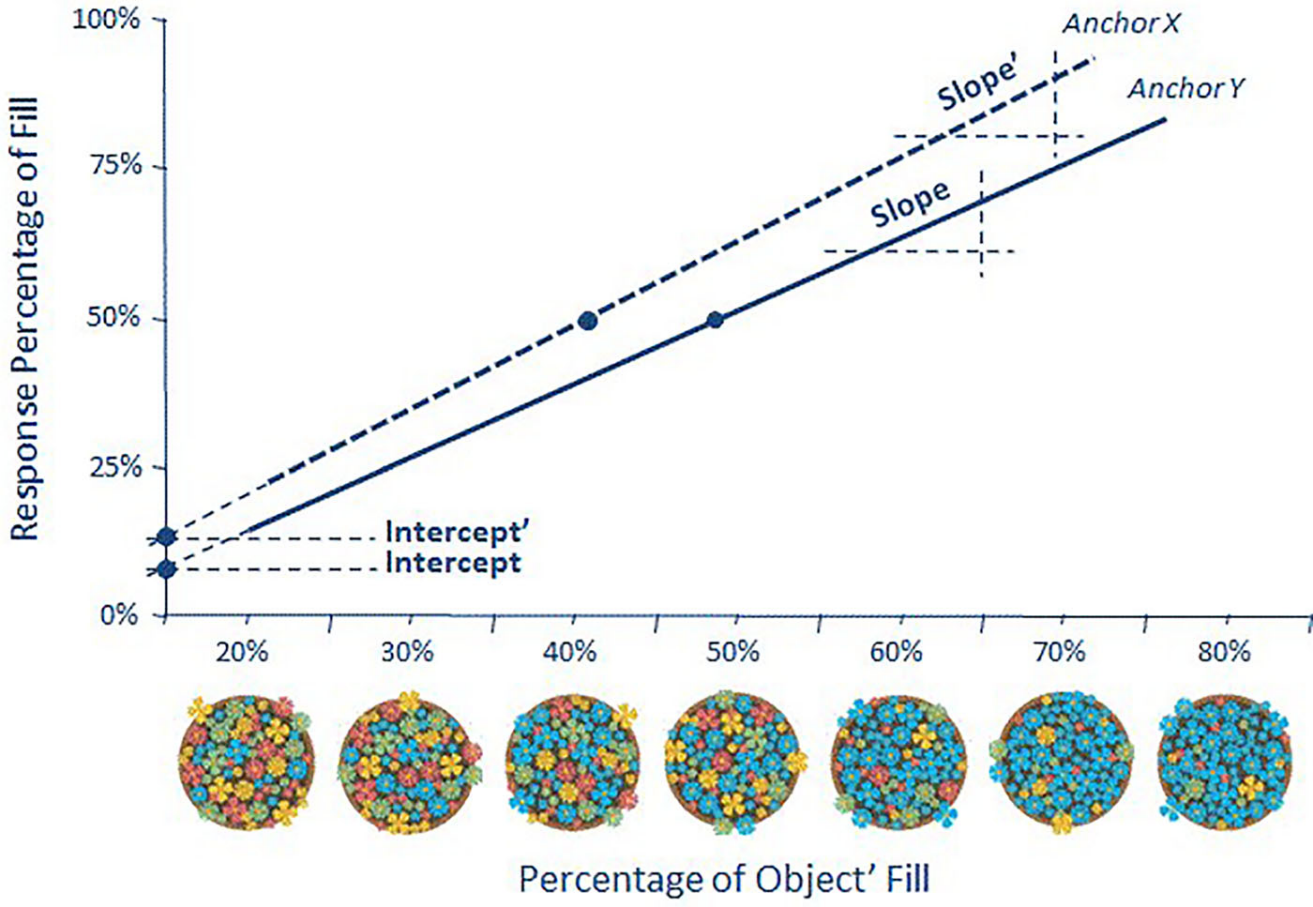
Two examples of a mapping of each absolute estimate for the linear function of each anchor. The difference in the point of subjective equality (PSE) for each anchor function represents differences in the calibration that individuals made for the estimates of each image, and so the component bias. The difference in the difference limen (DL) or slope (i.e., the increment of estimation for each unit of the actual feature) quantifies the component of the effect associated with the sensitivity of the perceptive system to different feature percentages.

Figure 3 shows how the two indices are calculated and represents the anchoring effects we are expecting to find, namely that anchors change the PSE indices representing the bias component of the effect, but also it will promote changes in the DL representing the impact over perceptual sensitivity.

In the mapped linear function, the PSE is equivalent to the inverse of the average of the deviations between the subjective estimates and their respective identity points (the real magnitude). The PSE represents the point (of the feature percentage) where the individual provides a subjective value of 50%. Therefore, the PSE provides information about the degree of calibration of the subjectively perceived proportions (indexing the response tendencies), which can change if these response tendencies are moved by the presence of an anchor.

The sensitivity index, represented by the DL, is negatively related to the slope of the linear psychophysical function and provides information about how well the levels of the subjective dimension match the real proportion of the percepts. Therefore, these slopes are indices of sensitivity (see Church & Deluty, 1977; Macmillan & Creelman, 2004).

To gain a deeper understanding of the underlying processes behind individuals' estimates, we conducted further tests on the reaction times associated with these estimates. Our focus was specifically on the stimuli features that elicited greater uncertainty in participants' estimates under different types of anchors. Additionally, by analyzing reaction times, we aimed to explore the dynamics between the two‐stage phases and determine whether an absolute estimate was already formed or not before providing a relative estimate. This approach allowed us to examine the availability and timing of absolute estimations within the task.

**FIGURE 4:**
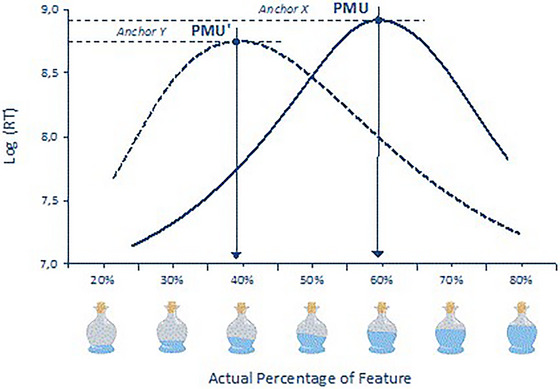
Two examples of inverted U‐shaped functions are represented (in the context of hypothetical X and Y anchors), mapping each logRT mean of relative judgments for each feature percentage. The inverted U‐shaped function represents the fact that logRTs increase with discrimination difficulty. A point of maximal uncertainty (PMU) identifies the feature percentage that, for each participant and each anchor, is the most difficult to discriminate.

For this testing, we propose to analyze logRT distributions, following Birngruber et al.’s (2015) suggestion of treating the actual stimuli level as bins (i.e., mean logRT as a function of feature percentage). These distributions are centered when there is a match between the anchor and the real proportion such that only 50% of targets are not restrained in the higher and lower values. The waveform moment analysis (Cacioppo & Dorfman, 1987) of these curves allows isolating for each “individual by anchor” cell the point where the participant felt maximal uncertainty (PMU), representing the feature percentage (in each anchor) at which the participant had more difficulty discriminating (see Figure 4).

We further explore the relationship between relative and absolute judgments of the estimation task, by assessing whether and how the differences in response tendencies or sensitivity that occur in the first phase (the classification decision made in the presence of the anchor) are subsequently transferred to the absolute judgments. We may rely on a complementary pattern on RT analysis in phases one and two to infer the likelihood of a specific response being made available in this first phase. Based on Weber's Law, we anticipate that larger differences between the information presented by the percept and the anchor will be more readily detectable than smaller differences. Smaller differences are likely to create uncertainty for participants when making relative judgments, prompting them to resort to an immediate absolute estimation. Consequently, participants are expected to exhibit faster response times in the second phase of the task as a result.

### 2.3 Overview of the study

In this overview, we summarize our goals and hypothesizes associated with the perceptual estimation anchor task.

Our first goal is to define assimilation anchoring at a perceptual level within the natural setting where perceptual estimation quantities are studied within a psychophysical approach. We expect to replicate the previous finding of changes in the central tendencies of individual estimates promoted by the presence of an anchor. However, we also expect to clarify how psychophysical functions are distorted by the presence of an anchor, by analyzing both the relative and absolute estimates made by each individual. As illustrated in Figure 3, the effect may be indexed as a general response bias (differences in the PSE associated with different anchors). However, we also expect evidence of a loss of sensitivity to feature percentages that are more distant from the anchor, also illustrated in Figure 3.

Our second goal is to document anchor effects already occurring when the anchor is activated, that is, when participants perform the relative judgment, as represented by the hypothesis illustrated in Figure 2. If participants are already anticipating the generation of absolute judgment (as suggested by Strack & Mussweiler, 1997), we should expect to find such evidence.

The third goal of this study is to test the relationship between anchoring effects and individuals’ uncertainty about the real proportion perceived in a target image. To accomplish this, we will also depend on analyzing the estimated reaction times.

Our final goal is to address the dynamics between the two phases, relating the anticipation of a response to a decrease in uncertainty. We expect that whenever individuals experience uncertainty in the first phase, if they can anticipate the generation of the absolute judgment that uncertainty must be reduced, responses will be faster in the second phase.

## 3 METHOD

### 3.1 Participants and design

Seventy‐five students (*M_age_* = 20.56 SD = 4.37; 85% female) participated in this study in exchange for credits. All participants estimated the percentage (i.e., proportion to the whole) of a visually presented feature that varied in seven levels (see details of the task above). These feature percentages were orthogonally associated with an anchor also varying in seven levels (a 7 × 7 within‐design).

### 3.2 Procedure

Participants performed the experimental task in the laboratory being conducted using E‐Prime 2.0 software (Psychology Software Tools, Pittsburgh, PA, USA) on a desktop computer with a 17‐inch screen. All stimuli (see above) were presented on a white background with black lettering. After being welcomed to the study, participants provided their informed consent and demographic information (age and gender). The within‐participants' design was operationalized by presenting to each participant 343 sets of two trials. Each set comprised one image category that was quasi‐randomly selected from all available categories. Seven exemplars were selected to be associated with each cell of the design: the crossed combination of seven levels of proportions and seven levels of anchors. The procedure ensured that no image category was repeated. This allowed for the complete counterbalancing of all possible combinations of materials, distributing the biases arising from the specific content of the images among the participants. Simultaneously, it prevented participants from encountering the same image multiple times.

The instructions for this task emphasized that participants would first need to estimate whether the feature percentage was higher or smaller than a randomly selected value (referred to as the anchor, ranging from 20% to 80%). Subsequently, participants were asked to estimate the precise value of the feature percentage presented. Given the substantial number of judgments required, it was expected that participants' responses would be spontaneous, reflecting their best guess in each instance. Participants learned in advance that each trial consisted of two screens—both displaying the image and a question below it. There was no separation between trials such that after responding to one question the next screen was immediately presented. On the first screen, the question was *Is the percentage of “…”* (the entity associated with the image—see Figure 1—such as “*blue flowers*” or “*lighted windows*”) (←) below X or (→) above X, where X is the selected anchor. To provide this relative judgment, the participant pressed the arrow ← (for below) or → (for above). On the subsequent screen, the question changed to *What is the percentage of…*, and participants were asked to type their absolute estimates. For both tasks, participants were given the freedom to take the time they felt necessary to provide their responses, and this response time was recorded for future analysis. At the conclusion of the tasks, which typically took approximately 50–55 min, participants were thanked for their participation and dismissed.

### 3.3 Dependent measures

For both the relative and absolute judgments, RTs and psychophysical functions were analyzed.

#### 3.3.1 RTs and psychophysical functions

For each of the 49 cells of the design (7 percentages x 7 anchors), we calculated the proportion of “larger responses” of the relative judgments, the means of the absolute estimates, and the means of the log‐transformed RTs (RTs with SD > 3 for each participant were excluded) for each type of judgment. From the distributional analysis of each type of response, a PSE (indexing bias) and a DL index of response sensitivity were calculated for each participant x anchor (see Figures 2 and 3).

#### 3.3.2 Point of maximal uncertainty

The analysis of the RTs associated with the relative and absolute judgments allows us to isolate the point where the participants felt maximal uncertainty (PMU) in providing their estimates for each “individual by anchor” cell (see Figure 4).

## 4 RESULTS^1^

### 4.1 Relative judgments

**FIGURE 5:**
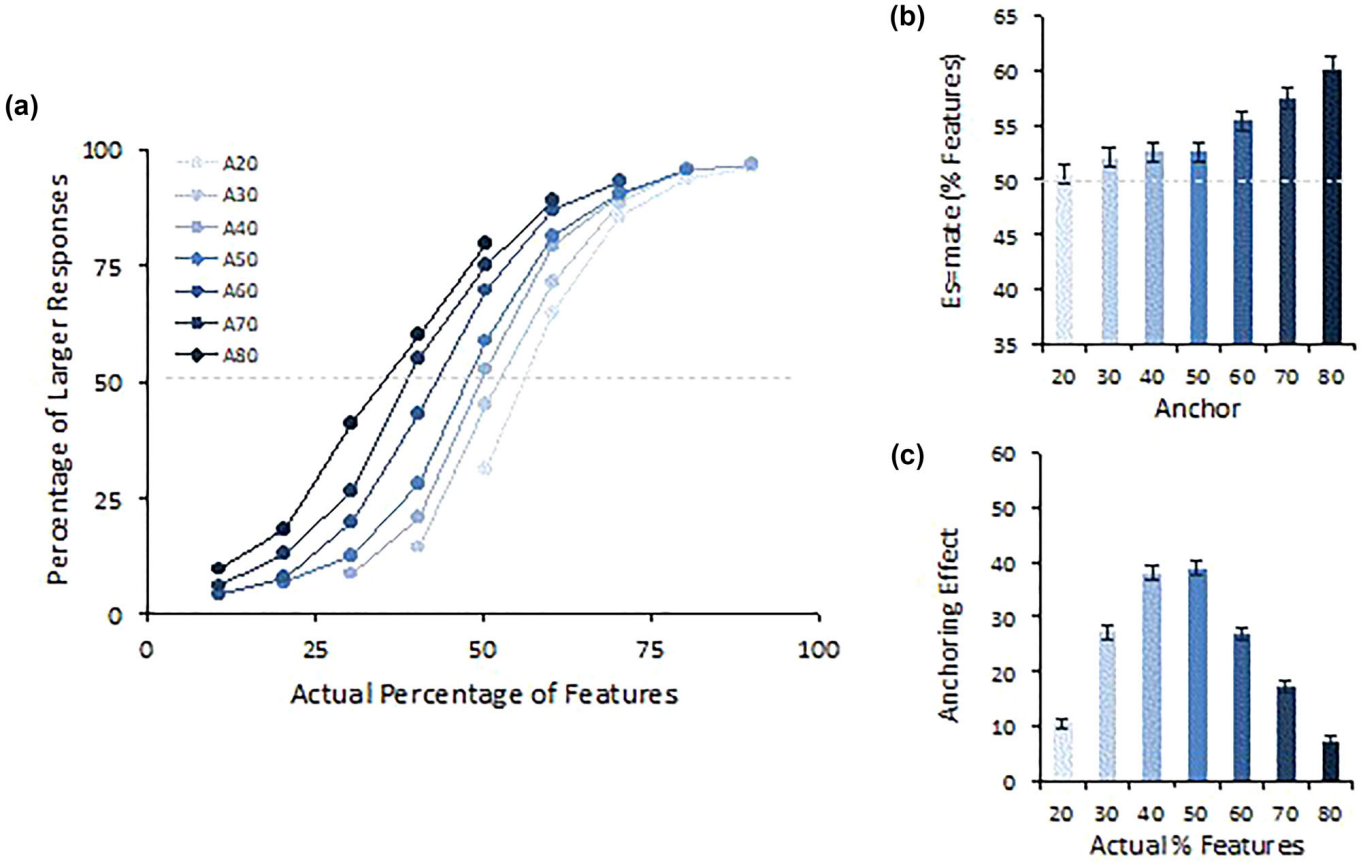
Individuals’ percentage of “larger” responses. (a) Interaction effects between anchors and feature percentage. Panel (b) represents the main anchoring effect. Panel (c) maps the magnitude of the anchoring effect represented by the linear contrast (−3, −2, −1, 0, +1, +2, +3) at different feature‐percentage levels.

The proportion of “larger” (than anchor) relative judgments was first analyzed using a GLM having the actual feature percentage and anchor level (7 × 7) as the within‐participant levels. The results are graphically represented in Figure 5, showing that the interaction component was significant, *F*(36, 2664) = 58.02, *p* < .001, *η_p_^2^ =* .49 (panel (A)^2^, and the two main effects. To understand the pattern of the main effect of feature percentage, *F(*6444) = 2202.96, p < .001, η_p_^2^ = .97, and the main effect of the anchor level, *F*(6444) = 511.33, *p* < .001, *η_p_^2^ =* .87, see panel (B). These results clearly show that the anchors were already biasing individuals' decisions in this phase.

To understand which actual percentages the anchoring effects were stronger for, we conducted further analysis on the interaction component. For this, we made the anchoring effect to be represented by the linear contrast (−3, −2, −1, 0, +1, +2, +3). Panel (C) of Figure 5 clarifies that anchor effects increase when the stimulus is most ambiguous (near the 50% or actual feature percentages).

**FIGURE 6:**
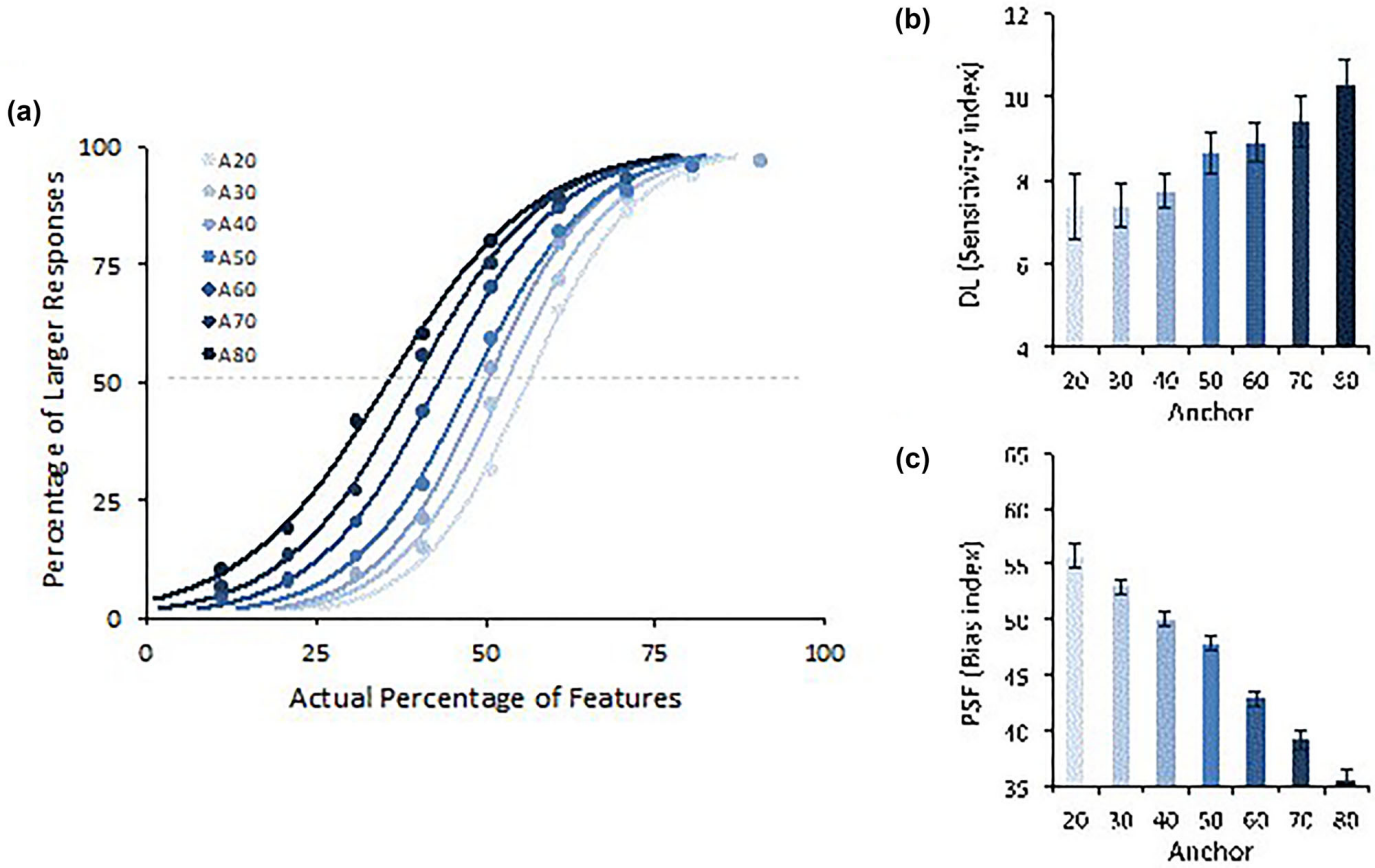
Psychophysical analysis of relative judgments: (a) Mean individual logistic psychophysical function. (b) The impact of the anchor on individuals' ability to discriminate quantities (DL). (c) Anchoring effects as a bias (PSE). DL, difference limen; PSE, point of subjective equality.

Following a psychophysical approach, a logistic function was fitted to each anchor for each participant using the GraphPad Prism software (La Jolla, CA). A summary of these individual logistic psychophysics functions (all having a good fit; *R* ^2^ > 89%) for each anchor is represented in Figure 6. For each participant x anchor function, the PSE and the DL were calculated. Panel (C) of Figure 6 illustrates how the PSE varies with the anchor level *F*(6444) = 109.87, *p* < .001, *η_p_^2^ =* .60, suggesting that the higher the anchor, the greater the number of “larger responses” that occur (lower values of the PSE indicate a stronger positive bias). Panel (B) shows the impact that the anchors exert on the individuals' ability to discriminate quantities, *F*(6444) = 4.33, *p* < .001, *η_p_^2^ =* .06, showing that the higher the anchor, the lower the sensitivity (indexed by higher DL). Namely, when the anchor is a low value, individuals tend to respond more systematically to all target percentages than when the anchor is higher. When the anchor becomes higher, individuals tend to respond more discriminatively and, so, are more dependent upon the real proportion of the stimuli.

Taken together, these results clarify that the impact of an anchor is much more than one of creating a response tendency (i.e., changing the PSE indices) since it also changes the sensitivity to differences in the magnitude effect (i.e., impacting the DL indexes).

### 4.2 Absolute estimates

**FIGURE 7:**
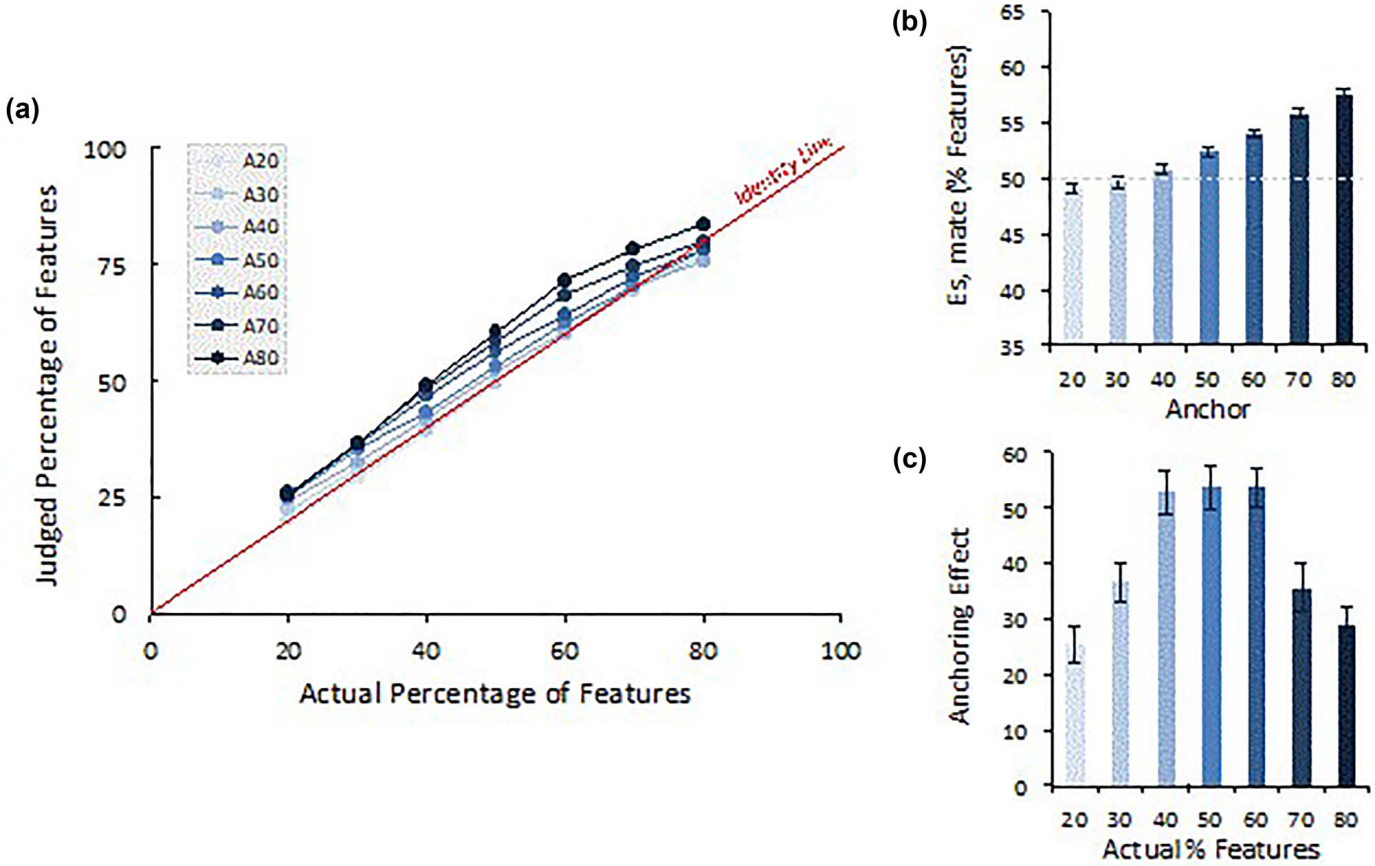
General linear model analysis of means of absolute estimates (judged feature percentage) for anchoring and feature percentage. Panel (a) plots the absolute estimates within the 7 × 7 cells; (b) the main anchoring effect; panel (c) maps the anchoring effect represented by the linear contrast (−3, −2, −1, 0, +1, +2, +3).

Mean percentage estimates were analyzed using a GLM approach (repeated measures 7 × 7 ANOVA). As expected, (Figure 7), both feature‐percentage *F*(6444) = 3492.61, *p* < .001, η*p* ^2^ = .98 and anchoring effects *F*(6444) = 138.86, *p* < .001, *η_p_*_2_ = .65 (see panel (B)) emerged. These results make clear that estimates increase with the increase in the anchor, but importantly, they also match the real feature percentage. That is, they adjust to reality. The interaction effect was also significant, clarifying that the magnitude of the anchoring effects is dependent on the target real percentage, *F*(36,2664) = 5.93, *p* < .001, *η_p_*_2_ *=* .07) (see panel (A)). To better understand this modulation of the anchor effect, we defined anchoring by the linear contrast, −3, −2, −1, 0, +1, +2, +3, and mapped it to panel (C) of Figure 7.

This analysis shows that the strength of anchoring effects being dependent on the image feature percentage is higher for more ambiguous quantities (feature percentage of approximately 50%) and lower for more extreme values.

**FIGURE 8:**
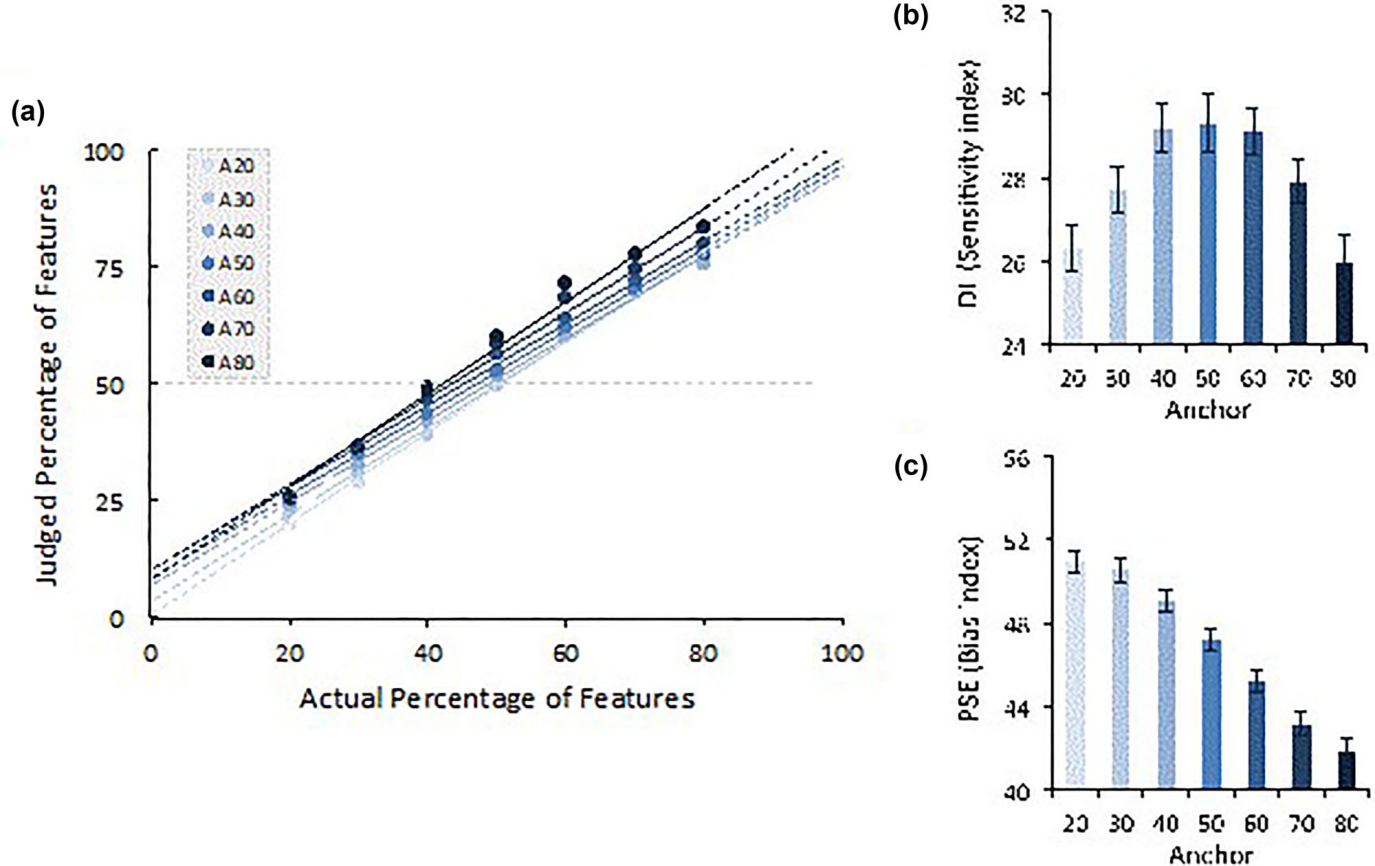
Panel (a) shows the summary of the individual linear psychophysical function made to each participant's mean estimate (judge feature‐percentage) associated with different anchors. Panel (b) plots the impact that anchors exert over sensitivity indexes (DL). Panel (c) plots anchoring effects of the bias indexes (PSE). DL, difference limen; PSE, point of subjective equality.

We further address the absolute estimates within a psychophysical approach. A linear function was fitted to each anchor for each participant, which allowed us to calculate PSE and DL indexes from slope and intercept (abscissa = 0) parameters. A summary of these individual psychophysics functions (mean: 94% of good fit) for each anchor is represented in Figure 8. The analysis of each psychophysical function allowed us to isolate the bias promoted by the anchor and its effects on individuals' capacity to discriminate perceptual quantity differences. Individual PSEs and DLs for each of the seven anchors were subsequently analyzed within a repeated measures analysis. Both effects were significant. Anchor impacts the PSE parameters, *F*(6444) = 111.41, *p* < .001, *η_p_^2^ =* .60, suggesting anchors' bias response tendencies, with a general tendency that higher anchors produce higher judgments (lower PSEs) (see panel (C) of Figure 8). Anchor effects also occur for the DL parameters, *F*(6444) = 10.46, *p* < .001, *η_p_^2^ =* .12, suggesting that anchors exert an impact on individuals' ability to discriminate quantities (panel (B) of Figure 8). Interestingly, this analysis suggests that the impact of anchors on sensitivity to the actual percentage of features depends on the extremity of the anchor. When the anchors are moderate compared to extreme anchors, individuals experience a notable decrease in their ability to accurately estimate the true proportion of the features being presented, resulting in higher DL values.

### 4.3 Reaction times

**FIGURE 9:**
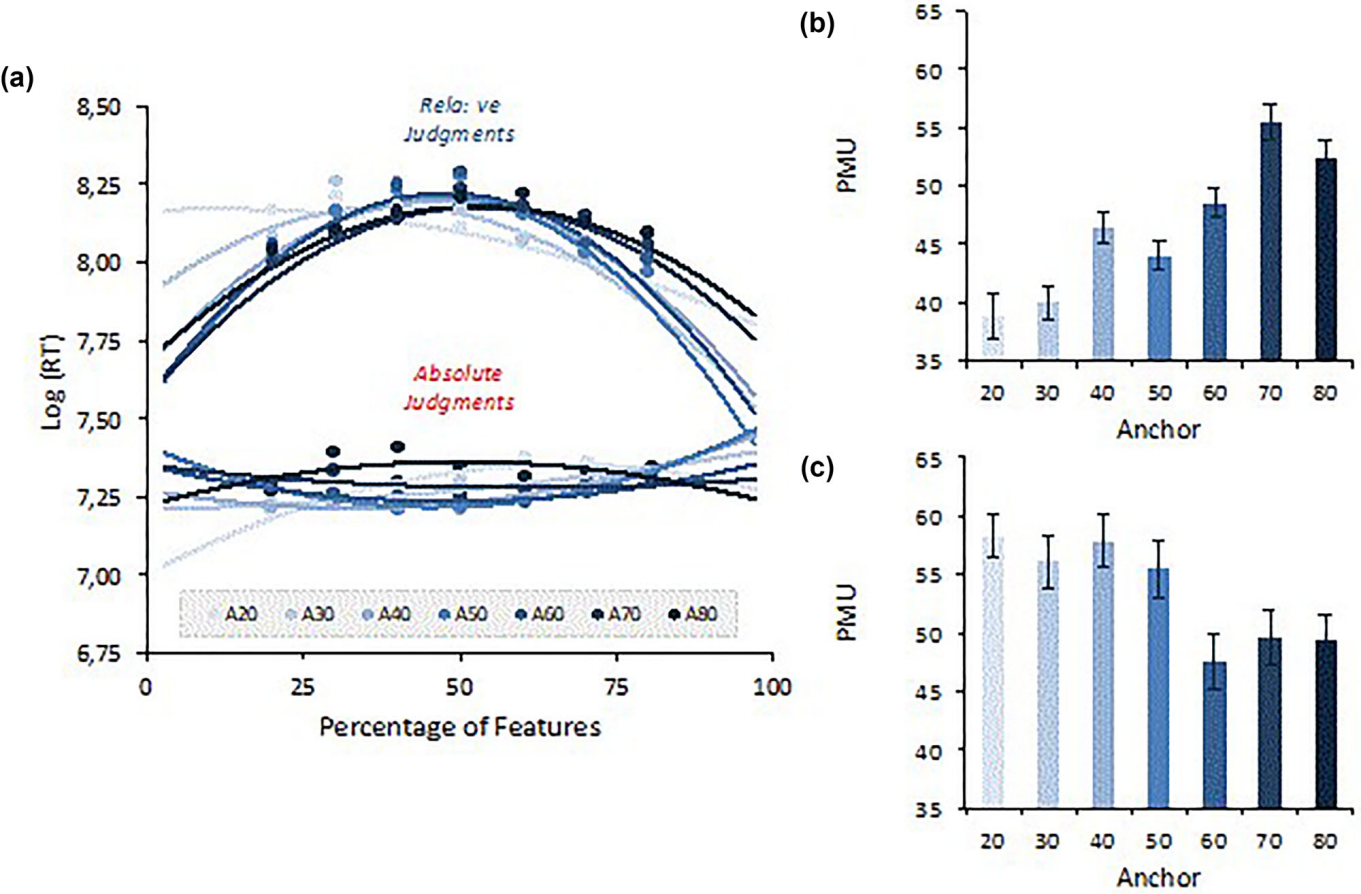
(a) Polynomial fitting of RT distributions for relative and absolute judgments (fitting of overall curves). Anchors are shown to modulate the difficulty (higher logRTs) with which different feature percentages were estimated. Panel (b) shows anchor impact on PMU for relative judgments. Panel (c) shows the anchor impact on PMU for absolute estimates.

A polynomial fitting of RT distributions for relative and absolute judgments (fitting of overall curves) is presented in Figure 9. Higher logRTs suggest a greater difficulty in estimating the real percentage presented in each target image.

The analysis of the relative judgments suggests, as expected, that the logRT distributions for relative estimates show that the 50% feature percentage is the most demanding condition (longer logRTs). The PMU values were calculated for each anchor and analyzed in a repeated‐measures ANOVA. The results show that PMU increases as a function of anchor for relative judgments, *F*(6444) = 17.13, *p* < .001, *η_p_^2^ =* .19. That is, more time is taken to respond when a value is higher or lower than when the anchors are higher (see panel (B) of Figure 9)

The analysis for absolute estimates shows an inverse pattern to one found for relative judgments. It is when real proportions are more difficult to deal with in the first phase (50%) that they become easier to deal with in the second phase. It is in the 50% conditions that absolute estimates take less time, suggesting that participants are likely relying on a previously made estimation, underlying the relative judgment when making the absolute estimates. Anchors also seem to modulate the difficulty with which different feature percentages were estimated, illustrating that higher anchors yield lower PMUs, while lower anchors yield higher PMUs, *F*(6444) = 4.15, *p* < .001, *η_p_^2^ =* .05, but now with an inverse pattern than the one occurring for the relative judgments (see panel (C) of Figure 9). The different impacts of anchors on the PMU of relative and absolute judgments are highly informative. More specifically, the difficulty felt in providing a binary decision (i.e., relative judgment) is higher when the anchor is similar to the feature percentage. However, when providing the subsequent absolute estimate, anchors lower than 50% make the estimation of a value above 50% more difficult, whereas anchors above 50% make the estimation of values below 50% more difficult.

## 5 DISCUSSION

Our results strongly suggest that perceptual anchoring effects concerning proportion estimates occur as systematic interferences over two components of each individual's psychophysical function. Anchors both affect response bias (criteria) and modulate quantity sensitivity. Importantly, neither of the biases prevents individuals from having a good perceptual and estimate performance, adjusting their responses properly to the features of the stimuli.

Additionally, the results show that anchor impacts more than the absolute estimate usually addressed in the literature. Within a two‐phase paradigm, we show evidence of anchoring over relative judgments both as a biased tendency and a modulation of sensitivity to the real proportions being perceived. Perhaps more relevant for supporting a better understanding of the phenomena, our data show that anchors change the contextual feature where individuals perceive more uncertainty. As expected, anchoring effects relate to the context of uncertainty. The effects were stronger for percentages of approximately 50%, exhibiting the same curvilinear shape found by Wegener et al. (2001). Extreme values, likely because they are less ambiguous, reduce the effect magnitude of the anchoring effect (see Jacowitz & Kahneman, 1995; Nisbett et al., 1981). However, anchors change the feature percentage for which participants experience more uncertainty. The effects were stronger for percentages around 50%, exhibiting the same curvilinear shape as found (Moyer & Landauer, 1967). For these relative judgments, data show that higher anchors increase the PMU and lower anchors decrease the PMU.

However, an important feature of the process is that the uncertainty that is experienced by participants when they first meet the actual feature proportions seems to be resolved by asking participants for a relative judgment. There is no evidence of an experience of uncertainty about the same value when participants are subsequently asked to make an absolute judgment. Now, more uncertainty is experienced concerning higher feature percentages, and the effects of anchors over the PMUs become different. This RT analysis of both judgments suggests that an absolute response is made available in the first phase (yielding higher RTs) and further employed in the subsequent phase (yielding lower RTs).

### 5.1 Relevance of the findings

As we argued in the introduction of this paper, the findings provided in this study extend perceptive/physical anchoring research, clarifying how the effect is mapped in our psychophysical functions. As such, it has potentially interesting theoretical implications for the account of those effects.

Importantly, our analysis of the theoretical relevance of these data for current alternative explanations of anchoring effects assumes a multiprocess view (e.g., Simmons et al., 2010; Wong & Kwong, 2000), so that in our data, the anchor does not bias a memory process. For those not convinced that anchoring is a multiprocess phenomenon, our data should be considered a challenge to memory accessibility accounts (e.g., Mussweiler & Strack, 2001) since it is not obvious how it would deal with estimations of perceptual quantities that do not require selective retrieval of exemplars.

From those studies that first address anchoring occurring at a perceptual level (see above), only LeBoeuf and Shafir (2006, 2009) address their theoretical explanation. They rely on the insufficient adjustment heuristic (e.g., Tversky & Kahneman, 1974) to account for the perceptual anchoring effects. Our study offers several insights for anchoring and adjustment accounts. By providing a systematic variation of anchors and perceptive reality, our approach indicates that, although anchors interfere with individuals’ ability to estimate perceptive proportions, their judgments match the variability of reality. Given the evidence of anchoring, the most likely possibility for this is that they adjust their initial estimates to the actual feature percentage, although not as much as necessary. Additionally, supporting the claim that the same level of adjustment to the perceived quantity occurs, the anchor effects are reduced when individuals have better access to the real proportions and higher when conditions are of greater uncertainty. Although this evidence for insufficient adjustment is indirect, this methodology may allow for more direct observation of this adjustment process. However, for this claim to account for the anchor effects in the levels of sensitivity found for absolute judgments, extra assumptions would have to be made. Namely, instead of simply assuming that individuals anchor their judgment at a point of the scale and that an insufficient adjustment occurs, the explanation would have to assume that the magnitude of those adjustments is dependent on the magnitude to be estimated and that the anchor also changes the perception of the magnitude. To clarify these points, future studies could address estimations for which a psychophysical approach already provides information that a distortion (not promoted by the anchor) will occur. In this regard, researchers could address proportional judgments that are either below 20% or above 80%, for which estimations are known to be biased regardless of the presence of an anchor (e.g., Sheridan & Ferrell, 1974; Varey et al., 1990; Wickens, 1992). Alternatively, research could focus directly on numerosity estimates, known to have a clear inflection in the number estimation function at approximately 20 (Portley & Durgin, 2019). Scale distortion theory (Frederick & Mouchon, 2012; Mouchon & Frederick, 2013) could offer an alternative account for our results. According to this account, anchors would contribute to changes or distortions in the response scale, making the anchors closer to the center of the scales. As such, the approach should expect the anchor to interfere not only with the absolute judgments’ bias/intercept but also with the changes in scale sensitivity. This approach considers relevant the distance between anchors and stimuli, seeing wider differences promoting the encoding in terms of coarser scale units and, consequently, less sensitivity (a so‐called rubber rule scale effect, see Lawless et al., 2000; Riskey et al., 1979). Evidence of a loss in sensitivity for extreme values or extreme anchors should be expected in our data. This pattern of results was only found for the extremity of anchors on relative judgments and not for absolute judgments. Only further research can clarify whether absolute judgments not preceding relative judgments would show similar effects to better clarify the relevance of the data to the scale distortion approach.

Oppenheimer et al.’s (2008) priming proposal suggests that anchors activate general notions of “largeness” or “smallness.” Authors sustain the idea on data showing cross‐modally anchoring effects (when the anchor cannot prime a number, cannot serve as the basis for adjustment, or activate memory information). There are some relevant aspects of this possible explanation of our data. First, by defining the anchor, not as an absolute value but as a relative value (the anchor is a smaller than or a larger than) number, we may be stressing the relevance of the first phase of the two‐phase paradigm for the task. Second, by assuming that a categorical dimension (small vs. large) overlaps with a continuous dimension (the one to be estimated), the approach adapts to the idea that anchor impact occurs both as a bias and as a change in sensitivity. Within this perspective, the relative judgments show evidence of an assimilative bias—that is, larger anchors will increase “larger” responses—and this would prime larger absolute judgments. However, this approach does not explain why the anchoring effects occur immediately on relative judgments and with the pattern observed in our data.

Our analysis of RT distribution, following Birngruber et al. (2015), suggested treating feature percentages as bins and offered evidence that anchoring is highly sensitive to uncertainty. Our results offer further support to the hypothesis that anchoring effects are more likely to occur when uncertainty is experienced (less extreme proportions). However, it also adds to the fact that anchors interfere with the context where individuals experience such uncertainty. Future studies can address how this impact is related to the fact that anchors change our sensitivity to real proportions. In our view, none of the previously reviewed explanations of the phenomena help us to understand this anchoring effect over our experience of uncertainty. Future studies can also use RTs within our multiple‐trial paradigm to follow other new approaches to anchoring, such as by using a mental chronometry framework (e.g., Sternberg, 1966). In particular, the additive and interactive effects between anchoring manipulation and other factors may be able to reveal the precise processing stages of the anchoring manipulation.

In addition, our results offer stronger empirical support to the claim made by Strack and Mussweiler (1997) that within a two‐phase paradigm, an absolute response is already made available in the first. Our RT analysis of both judgments makes clear that an absolute response is made available in the first phase and further employed in the subsequent phase, establishing an inverse relationship between conditions that lead to higher RTs in relative and absolute judgments. Additionally, it suggests that by considering only specific processes engaged in the second phase, researchers may lack access to the real mechanisms promoting the anchoring effects.

We believe that our approach offers a highly methodological innovation to the field of anchoring. This approach allows the use of signal detection theory to better characterize the type of bias exerted by anchors and address the dynamics between relative and absolute judgments. We believe that even without a psychophysical approach, some features of this new methodology can be extended to study anchoring phenomena that are driven by memory biases. The most relevant feature is the manipulation of anchor levels as a within‐factor, involving the definition of several levels of an anchor. The literature has already demonstrated that numerical anchors are easily manipulable in such contexts. This allows us to access memory anchoring within a repeated trial setting, enabling the utilization of a signal detection approach to separate the two components of the effect. Through this design, we can investigate how variables known to influence memory accessibility or anchoring effects, such as knowledge, familiarity, attitudes, and emotions, impact responses by either reducing discriminability or imposing a response bias. The possibility of various factors contributing to the effect through different pathways may help explain why certain factors (see the review of Furnham & Boo, 2011).

## 6 CONCLUSION

This paper establishes a psychophysiological methodology to show evidence of anchoring effects at a perceptive level. By accessing estimates of several events varying in feature percentage in the presence of different anchor levels, our results show that anchors not only bias individuals' response tendencies but also interfere with individuals' sensitivity to stimulus differences. Anchoring also changes the level of feature percentages where individuals experienced higher levels of uncertainty when making those estimates. Finally, this study states the relevance of the usually disregarded relative‐judgments phase of a two‐phase paradigm, showing not only that the relative judgments are also biased by anchor levels but also by suggesting that, to make those relative judgments concerning uncertainty features, individuals have to make an absolute estimate, making it available subsequently. Together, these results provide new challenges to the currently available explanations of the anchoring effect.

## AUTHOR CONTRIBUTIONS

The first author conceived the idea, and both authors made equal contributions to designing and implementing the research, as well as formulating the data analysis plan. The second author was responsible for conducting all result analyses and creating the figures. Both authors collaborated on manuscript writing and discussing the results.

## CONFLICT OF INTEREST STATEMENT

The authors have no conflicts of interest to declare.

### PEER REVIEW

The peer review history for this article is available at https://publons.com/publon/10.1002/brb3.3254.

## Data Availability

The data that support the findings of this study are openly available at https://osf.io/v7b8y/?view_only=665f89ccb91a412fb670627b72937e53

## Notes

1 All the data represented in the figures presented in this section can be consulted as tables in the Supporting Information here : https://osf.io/v7b8y/?view_only=665f89ccb91a412fb670627b72937e53

2 These functions do not possess the same intercepts, since, for instance, there is no “larger response” associated with higher anchors when image proportions are lower, and vice versa.
